# Ligands of Acid-Sensing Ion Channel 1a: Mechanisms of Action and Binding Sites

**Published:** 2019

**Authors:** D. B. Tikhonov, L. G. Magazanik, E. I. Nagaeva

**Affiliations:** I.M. Sechenov Institute of Evolutinary Physiology and Biochemistry Russian Academy of Sciences, Thorez pr. 44, 194223, St.Petersburg, Russia

**Keywords:** CNS, ASIC, potentiation, inhibition, mechanisms of ligand-receptor interactions, binding sites

## Abstract

The proton-gated cationic channels belonging to the ASIC family are widely distributed in the central nervous system of vertebrates and play an important role in several physiological and pathological processes. ASIC1a are most sensitive to acidification of the external medium, which is the reason for the current interest in their function and pharmacology. Recently, the list of ASIC1a ligands has been rapidly expanding. It includes inorganic cations, a large number of synthetic and endogenous small molecules, and peptide toxins. The information on the mechanisms of action and the binding sites of the ligands comes from electrophysiological, mutational and structural studies. In the present review, we attempt to present a systematic view of the complex pattern of interactions between ligands and ASIC1a.

## LOCALIZATION AND FUNCTION OF ASIC IN THE CENTRAL NERVOUS SYSTEM

Proton-induced ionic currents were discovered in 1980 by Krishtal and Pidoplichko [1], who suggested that reduced pH in an extracellular medium would trigger a population of proton-gated ion channels. Cloning of proton-gated ion channels in the mid-1990s classified them as a new family (ASICs – acid-sensing ion channels) belonging to the superfamily of degenerin/epithelial sodium channels (DEG/ENaC) [2]. The genes encoding ASICs have been identified in many vertebrate species, starting with *cyclostomata*. Four mammalian *accn1-4 *genes encoding at least six different subunits (ASIC1a, ASIC1b, ASIC2a, ASIC2b, ASIC3, and ASIC4) are currently known. These subunits can form both homo- and heterotrimeric complexes.

Proton-induced currents can be found in almost all types of neurons. The levels of expression of different ASIC subunits differ markedly depending on localization. Thus, ASIC1a, ASIC2a, and ASIC2b subunits are mainly found in the brain [3-5], while ASIC1b and ASIC3 subunits are more common in sensory neurons of the spinal cord and spinal ganglia [6, 7]. Ca^2+^-permeable ASIC1a, which are similar to the ASIC1a receptors of hippocampal neurons in terms of their functional and pharmacological properties, were found on the surface of NG2 hippocampal glial cells at a high density [8]. In the hippocampus, ASICs predominantly reside in interneurons, while the proton-induced currents in CA1 pyramidal cells are negligible [9]. In the central nervous system, ASICs are putatively involved in the mechanisms of synaptic transmission and synaptic plasticity, as well as many other systemic functions, such as memory and learning [10], fear and depression [3]. Their role can be determined by analyzing the mechanisms of drug addiction [11] and the pathogenesis of a number of mental disorders [12].

Taste, auditory, and photosensitive receptor cells [13-15], as well as smooth muscle cells lining the vessel walls [16], express ASICs on their surface, although to a much lesser extent than neurons. In the peripheral nervous system, ASICs are responsible for the perception of the pain stimuli accompanying inflammation, fractures, tumors, hematomas and postoperative wounds. Furthermore, they participate in mechanoreception [17]. Tumor growth also stimulates ASIC expression [18].

Most of the data pertaining to the physiological role of proton-gated ion channels are indirect, since they are based on the experimental findings obtained from knockout animals. Elimination of particular ASIC subunits allowed researchers to trace their role in the occurrence of certain behavioral phenotypes, as well as the involvement of ASICs in the development of various pathological processes in the nervous system [19].

Most of the data pertaining to the physiological role of proton-gated ion channels are indirect, since they are based on the experimental findings obtained from knockout animals. Elimination of particular ASIC subunits allowed researchers to trace their role in the occurrence of certain behavioral phenotypes, as well as the involvement of ASICs in the development of various pathological processes in the nervous system [19].

It was not until recently that direct evidence of ASIC involvement in synaptic transmission was obtained. The content of synaptic vesicles has pH ~ 5.2–5.7 [20]. Accordingly, as the vesicle content is released, pH of the synaptic cleft may transiently decrease by 0.2–0.6 pH units and lead to activation of both pre- and postsynaptic ASICs [21-23]. However, the contribution of ASIC-mediated postsynaptic currents is 15–20 times lower than that of glutamate-mediated postsynaptic currents [11, 24, 25].

Among the proton-gated channels expressed in the CNS, ASIC1a is the one most sensitive to acidification of the medium [26]. In addition, PcTx1, a specific inhibitor of ASIC1a homomers, eliminates most of the proton-induced currents in hippocampal and cerebral cortex cell cultures [27, 28]. Thus, most proton-induced currents in the brain are likely to be mediated by ASIC1a and ASIC1a-containing heteromers. These facts explain the keen interest in the properties of ASIC1a and their ligands.

The main challenge faced by neurophysiologists studying ASICs consists in resolving the contradiction between the obvious role of these channels in physiological and pathological processes and the small values of ASIC-mediated currents observed experimentally upon synaptic transmission. Meanwhile, the *in vitro *experiments involving CNS neurons demonstrate that the currents caused by acidification of the extracellular medium and application of glutamate, the major excitatory neurotransmitter, have similar amplitudes. The second problem is that ASICs expressed in the central nervous system desensitize (i.e., lose their ability to conduct current) rapidly in response even to minor acidification. Thus, acidification to pH 7.0 causes about 80% desensitization of ASIC1a and actually turns off their function [26].

The existence of endogenous compounds that modulate the function of ASICs under physiological conditions could be a solution to these problems. The NMDA-type ionotropic glutamate receptor is the classic example of such modulation. These channels can be efficiently activated only in the presence of a co-agonist, glycine [29]. That is why it is of great interest to search and study new ASIC ligands (and potentiators of ASICs in particular) in the context of both the fundamental problems of pharmacology and neurophysiology.

## ASIC1A LIGANDS

**Fig. 1:**
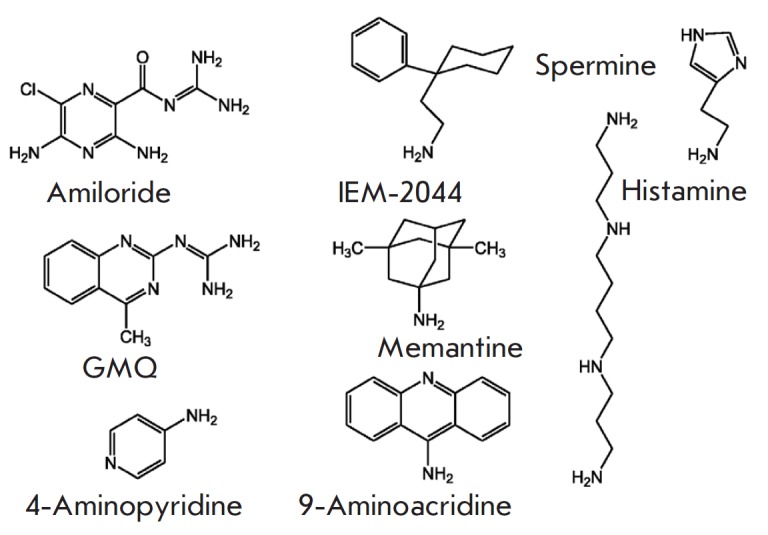
The chemical structures of some ASIC1a ligands

Studies focused on the relationship between the structures and molecular mechanisms of action of ASIC1a modulators are important for determining the physiological role of ASICs and designing a novel class of medications. Among ASIC1a modulators, there are synthetic compounds, endogenous organic substances and ions, as well as a number of peptide toxins from the components of natural venoms. The structures of some low-molecular-weight ligands are shown in *Fig. 1*.

**Amiloride**

Amiloride, a diuretic prescribed to patients with hypertension and heart failure, was the first discovered blocker of proton-gated ion channels [30, 31]. Amiloride acts as a nonselective ASIC blocker with low affinity to the binding site (IC_50_= 5–100 μM), which is also capable of blocking other ion channels and exchangers [32]. Interestingly, at higher amiloride concentrations its inhibitory effect is inverted to a potentiating or even activating action. Application of amiloride (EC_50_ = 560 μM) under neutral conditions (pH 7.4) activates homomeric ASIC3 and heteromeric ASIC3/ ASIC1b-channels and synergistically increases the currents through these channels in response to moderate acidification of the extracellular medium [33]. Hence, it is obvious that amiloride has a dual multidirectional effect on proton-gated ion channels.

**2-Guanidine-4-methylquinazoline (GMQ)**

The discovery of the unusual effect of amiloride on proton-gated ion channels has inspired researchers to synthesize a number of its analogs carrying the guanidine moiety and a heterocyclic ring [34, 35]. Among all these compounds, GMQ stands out for the specificity of its activating effect. It was the first synthetic nonproton activator of ASIC3; this fact suggests that there can be other synthetic/endogenous ASIC activators. At high concentrations (EC_50_ = 1 mM), GMQ can induce a stationary non-desensitizing current through the ASIC3 channels which is many times greater than the nondesensitizing current evoked by saturating concentration of the natural agonists (protons). The action of GMQ depends on extracellular Ca^2+^, being enhanced as the Ca^2+^ concentration in the medium decreases [36, 37]. In addition to its activating effect on ASIC3, GMQ also specifically interacts with ASIC1a. The effect involves a shift in the pH dependences of activation and steady-state desensitization towards more acidic values by approximately 0.2 pH units. Both effects have a competitive-like nature; i.e., both steady-state desensitization and activation develop completely, although at lower pH values [38].

**4-Aminopyridine (4AP)**

The well-known potassium channel blocker 4AP is another small molecule that can block ASIC1a homomers (IC_50_ ~ 760 μM) and heteromers containing ASIC1a, ASIC1b and ASIC2a subunits. As is the case for potassium channels, the binding site of 4AP in ASICs and other degenerin/epithelial channels resides within the pore, since its effect is significantly voltage-gated [39].

**Metal ions**

ASICs are inhibited by various metal ions [40–42]. It was shown that the affinity of protons to the channel directly depends on calcium concentration in the medium [43, 44]: the lower the Ca^2+^ concentration, the higher the affinity of protons is and, accordingly, the greater the ASIC responses are.

**Spermine**

Spermine is an endogenous polyamine that amplifies the proton-gated currents through ASIC1a and ASIC1a/2a channels [45]. The mechanism of ASIC1a channel potentiation consists of several components and involves a slowdown of inactivation (in other words, the activated channel remains open for a longer time); reduction of proton affinity to the receptor and, consequently, an increase in their responses at low background pH; and quicker channel recovery under repeated stimulation. All these effects enhance Ca^2+^ entry into the neuron through ASIC1a under ischemic conditions and, eventually, lead to cell death. Both blockade of endogenous spermine synthesis and blockade of the ASIC1a channels significantly increase neuronal survival in the *in vivo *and *in vitro *mouse models of ischemia [45].

**FMRF amides**

FMRF amides (Phe-Met-Arg-Phe-NH_2_), which prevail in the nervous system of invertebrates, as well as related peptides in the mammalian nervous system, activate some degenerin/epithelial Na^+^-channels [46]. They are unable to activate ASICs *per se *but can significantly potentiate the responses of ASIC1a and ASIC3 channels to the acidification of the medium [47, 48]. These peptides have a direct impact on the channel and slow down receptor desensitization, thus increasing the time for which the channel remains open after activation [49, 50]. These peptides also affect steady-state desensitization by shifting it towards stronger acidification [48]. Endogenous opioid neuropeptides, dynorphin and big dynorphin, also shift the steady-state desensitization and enhance the responses of ASIC1a upon weak acidification [51].

**Psalmotoxin-1 (PcTx1)**

The polypeptide toxin isolated from the venom of the South American tarantula *Psalmopoeus cambridgei *was the first-described specific inhibitor of ASIC1a channels (IC_50_ ~ 1 nM) [52]. PcTx1 consists of 40 amino acids and is formed by three antiparallel β-sheets twisted into loops, with a compact nucleus containing three disulfide bridges and residing in the center [53]. Psalmotoxin-1 inhibits ASIC1a channels by increasing receptor sensitivity to protons and shifting desensitization towards less acidic pH values [54]. Since ASIC1a are activated in response to a slight increase in proton concentration, even a small rise in the affinity of H^+^ to proton-binding sites is sufficient to switch receptors to the desensitized state. As a result, most of the ASIC1a becomes inactive in the presence of PcTx1 at pH 7.4, due to enhanced steady-state desensitization. The toxin preferably binds to the desensitized channel and stabilizes it in this state [55].

**MitTx**

MitTx was isolated from the venom of the Texas coral snake *Micrurus tener tener *in 2011 [56]. This peptide toxin resembles β-bungarotoxin in terms of its structure and consists of two non-covalently bound subunits. MitTx does not inhibit ASICs but activates both homo- and heterotrimeric channels [56, 57]. ASIC1a and ASIC1b homomers (EC_50_ ~ 9 and 23 nm, respectively) are the most sensitive ones to its action; ASIC3 channels are much less sensitive (EC_50_ ~ 830 nm). When applied together with a neutral solution, MitTx has virtually no effect on ASIC2a channels but strongly potentiates proton-gated currents through these channels by shifting the activation curve towards less acidic values.

**Mambalgins**

Mambalgins constitute a group of three toxins with a length of 57 amino acids. Two of them, mambalgin-1 and -2, different by only one amino acid at position 4, were isolated from the venom of the black mamba *Dendroaspis polylepis polylepis *in 2012 [58]. Mambalgin-3 was isolated from the venom of the green Mamba and got its name because it differs from the aforementioned two toxins only by the amino acid at position 23 [59]. All three peptides are structurally related to the three-finger toxin family, have similar pharmacological characteristics, and inhibit ASIC1a [59]. Mambalgin-1 inhibits ASIC1a via the following mechanism: it preferably binds to the closed channel and strongly shifts the pH dependence of activation to more acidic pH values. At the same time, the toxin moderately shifts the inactivation curve towards the alkaline region, thereby stabilizing the desensitized state of the channel and increasing inhibition [58].

**Hydrophobic monoamines**

Recently, the staff of the Laboratory of Biophysics of Synaptic Processes at the Sechenov Institute of Evolutionary Physiology and Biochemistry (Russian Academy of Sciences) has found that compounds with a simple chemical structure containing the hydrophobic/aromatic core and an amino group (hydrophobic monoamines) are modulators of native and recombinant ASICs [60, 61]. Among the four compounds tested in the first stage, only IEM-1921 exhibited no action against homomeric ASIC1a channels even at a concentration of 1,000 μM. The other three compounds, 9-aminoacridine (9AA), IEM-2117 and memantine, had a concentration-dependent inhibitory effect [60]. 9AA was the most active inhibitor. At a concentration of 1,000 μM, this compound, being co-applied with an activating solution with pH 6, caused 67 ± 8% (n = 6) inhibition. The action of 9-aminoacridine was pH-dependent: this compound at a concentration of 300 μM induced 80% inhibition at pH 6.8 and only 12% inhibition at pH 5.0. Therefore, the inhibitory effect of 9-aminoacridine is due to the shift in ASIC1a activation towards stronger acidification. A characteristic feature of memantine action was the abrupt acceleration of response desensitization. A similar effect of memantine on ASIC1b was demonstrated earlier [61]. A more detailed study of the mechanism of memantine action demonstrated that in this case the inhibitory effect is due to the open channel block. This conclusion is based on the fact that the effect of memantine was voltage-rather than pH-dependent [62].

Further structural and functional analysis [63] has identified potentiators of ASIC1a. We found that incorporation of a methylene group between the phenylcyclohexyl ring and the amino group in IEM-1921 conferred weak potentiation, while insertion of the second group enhanced potentiation (compound IEM-2044).

**Fig. 2:**
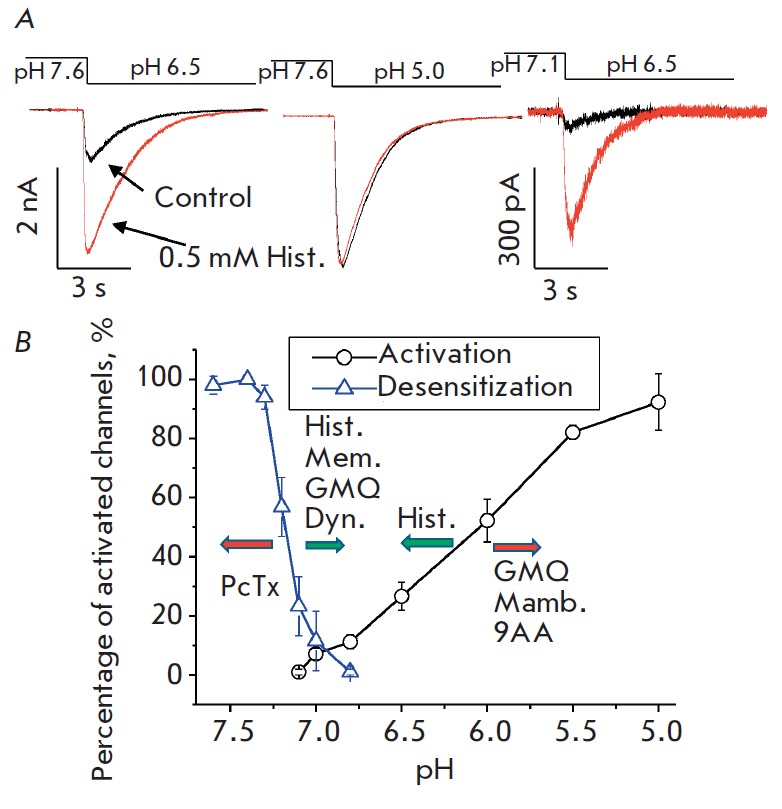
The mechanisms of action of ASIC1a ligands. *A *– the effect of histamine on the recombinant ASIC1a expressed in CHO cells. Histamine does not affect the maximal response (caused by a pH decrease from 7.6 to 5.0) but potentiates the partial response (pH decrease from 7.6 to 6.5), especially under conditions of partial desensitization (pH decrease from 7.1 to 6.5). B – pH dependences of ASIC1a activation and desensitization. The arrows show the directions of the shifts caused by ligands

The detection of potentiating activity for IEM-2044 has intensified further search for novel, potentially active drugs. The chemical structure of this compound is similar to that of histamine, a number of histamine receptor ligands and other endogenous amines, such as tyramine and tryptamine. Histamine [64] and its derivatives, alpha-methylhistamine and 1-methylhistamine [65], were shown to be strong and selective potentiators of ASIC1a. Potentiation takes place due to the shift in the activation curve, since the response to extreme acidification does not increase. Among the ligands of histamine receptors, this effect has been demonstrated for thioperamide and dimaprit. In contrast, compound A943931 caused inhibition that was dependent on the membrane voltage rather than on activating pH, which is indicative of a pore-blocking mechanism of action [65].

The initial study [64] did not reveal any effect of histamine on ASIC1a desensitization. However, a more detailed analysis showed that the effect of histamine increases at initial pH = 7.1 (i.e., under partial desensitization) (*Fig. 2*). A similar effect was also established for tyramine and tryptamine, which did not shift the activation curve [66]. Even memantine, an inhibitor of open ASIC1a channels, exhibited potentiating activity when applied between channel activations at pH 7.1 (i.e., in the case of interaction with closed and desensitized channels only) [65]. Therefore, different monoamines shift the steady-state desensitization of ASIC1a towards stronger acidification.

**Summarized data on the mechanisms of ASIC1a modulation**

It is difficult to establish clear relationships for the variety of data and hypotheses on the action of specific ASIC1a ligands. However, the accumulated large body of experimental data reveals some patterns.

According to a combination of features, some compounds can be classified as ASIC1a pore blockers. Amiloride, memantine and 4-aminopyridine are typical members of this group. Binding of these compounds in the pore leads to inhibition of ion transport independently of the degree of channel activation and desensitization. Blockers of cation channel pores are usually cations; in this case, their action depends on the membrane potential.

The second common type of ligand action consists in a shifting of the activation curve, which leads to either inhibition or potentiation of currents (*Fig. 2B*). Peptide toxin mambalgin and the low-molecular-weight compounds GMQ and 9AA shift the curve towards stronger acidification, while histamine ensures channel activation at higher pH values. A feature of this mechanism is that the ligands are potent only at low acidification levels, when ASIC1a activation is rather low. The activation curve can be shifted due to the allosteric effect on receptor affinity to protons or due to direct interaction with the proton-binding site. In the latter case, ligands of this type act as agonists or competitive antagonists, depending on the direction of action.

The third type of action is alteration of the steady-state desensitization of ASIC1a (*Fig. 2B*). Psalmotoxin is the best known example of compounds that enhance desensitization. Spermine and monoamines reduce desensitization, thus allowing ASIC1a to function under long-term acidification of media. A number of ligands, such as GMQ and histamine, simultaneously affect activation and desensitization. There is no correlation between these effects. Thus, GMQ shifts both curves towards deeper acidification, while histamine shifts the activation curve towards less significant acidification (*Fig. 2A*). Psalmotoxin, which is well-known as a desensitization promoter, may cause channel activation at alkaline pH values.

The question pertaining to the binding sites is even more complicated. Site-directed mutagenesis of the receptor is the method conventionally used to detect the ligand binding site. However, this approach does not provide unambiguously interpretable results. Mutations can either directly affect the ligand binding site (if the mutated amino acids reside in it), or allosterically modulate receptor affinity to the ligand by changing receptor conformation. In addition, mutations can significantly affect the functional properties of the channel, its activation and desensitization, which may further complicate data interpretation. In some cases, mutations result in complete loss of channel function. In this case, it becomes no longer possible to determine the effect of mutation on ligand binding. Thus, despite the high value of mutagenesis data, their structural interpretation requires great care. Therefore, the problem related to the ligand binding site can be solved only with allowance for the data on the ASIC structure and the molecular mechanisms of their activation and desensitization.

## ASIC STRUCTURE

**Fig. 3:**
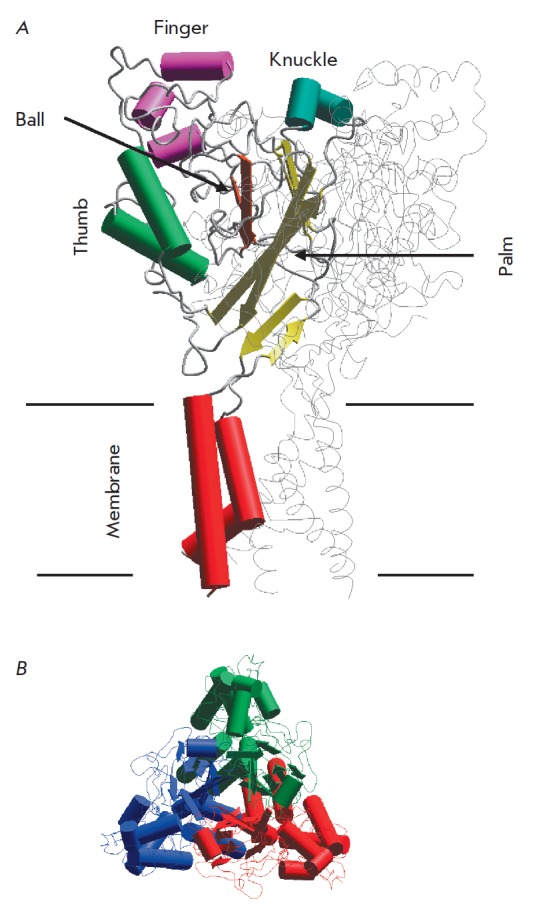
The general structure of ASIC according to X-ray crystallography. *A *– side view with the main parts in one subunit highlighted: Finger, Knuckle, Ball, Thumb, and Palm. *B *– the top view shows that ASIC is a trimer, in which the subunits (shown with different colors) surround the channel pore in the center

The first X-ray crystallographic structure of chicken ASIC1a reported in 2007 [67] has made it possible to establish the main elements of its structure (*Fig. 3*). The crystal structure of the functioning mutant with a lacking C-terminal region but retained N-terminal region and the portion required for channel opening was obtained later with a lower resolution (3 Å) [68]. Both proteins crystallize at low pH. Under such conditions, the ASIC1a channels exist in a desensitized state. Later, the ASIC1a structures were obtained in the open and closed states [69], making it possible to determine the activation and desensitization mechanisms.

ASICs are trimers whose subunits are symmetrically arranged around the central channel pore. The extracellular domain (ECD) of each subunit resembles a clenched fist attached to the transmembrane segments by a movable “wrist” [67]. Given this similarity, Jasti et al. [67] described the ECD in terms of a human hand holding a ball. Subsequently, this terminology has become commonly used as it turns out to be quite convenient. ECD can be divided into five subdomains: the palm, the finger, the thumb, the knuckle and the β-ball domains (*Fig. 3*).

An important feature of the ECD structure is the so-called acidic pocket, where several acidic amino-acid residues occupy a small area. It is located at a distance of 45 Å from the transmembrane region and is formed by interactions between the thumb-, β-ball, and finger domains of one subunit and a portion of the palm domain of the neighboring subunit. There are closely located three pairs of acidic amino-acid residues (Asp238-Asp350, Glu239-Asp346 and Glu220-Asp408) inside this pocket. The electrostatic repulsion between the negative charges of the side chains in these pairs of amino-acid residues retains the expanded conformation of the acidic pocket; so the channel is closed. Binding of the protons between carboxyl pairs takes place when the external medium is acidified, making the pocket take a more compact conformation. This causes conformational changes in the thumb domain, which in turn alters the wrist and the transmembrane domain. Hence, the acidic pocket is a site of the proton binding required for channel activation [69].

However, in addition to the amino acids in the “pocket,” there are several asparagine, glutamine and histidine residues in the lower portion of the palm domain (their p*K*a value also lies within the pH range that activates ASIC1a channels) [70]. In addition, complete removal of all three pairs of amino acids from the “pocket” dramatically reduces receptor sensitivity to protons but does not eliminate its ability to be activated in response to strong acidification of the extracellular medium [71]. In this regard, it is believed that several sites responsible for proton binding and further channel activation reside within one subunit.

The transmembrane segment of the ASIC domain is formed by six α-helices: two (TM1 and TM2) from each of the three subunits composing the functioning ASIC channel pore. The transmembrane segments of each subunit are involved in channel pore formation. TM2 directly lines the pore lumen, while TM1 plays a supporting role: it is in contact with the lipid bilayer and forms many bonds with TM2 of the same subunit, as well as with TM2 and TM1 of the neighboring subunit. Only a small C-terminal portion of TM1 directly lines the channel pore [72].

**Binding sites of ASIC modulators**

The most accurate data on ligand binding sites are obtained by X-ray crystallography and cryogenic electron microscopy. Several high-resolution ASIC structures in combination with such ligands as psalmotoxin, MiTtx and amiloride have been obtained to date [73, 74].

**Fig. 4:**
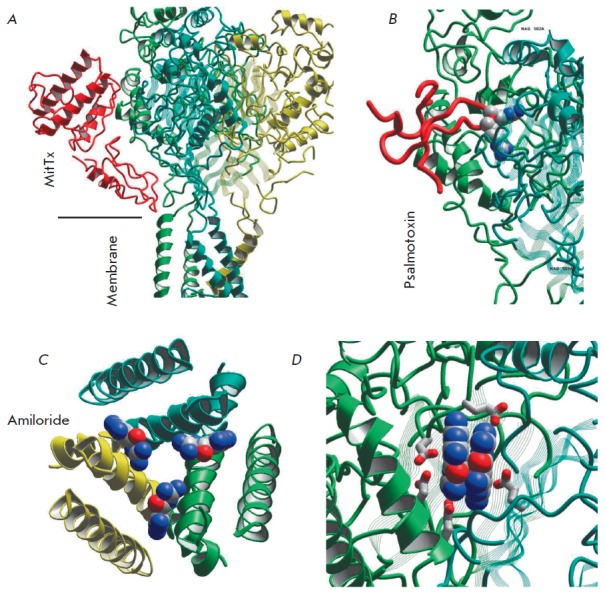
Binding sites of ASIC 1a ligands according to structural studies. *A *– MitTx that activates the channels and binds to the palm domain; *B *– psalmotoxin binds to the upper portion of the palm domain, and the central loop penetrates the acidic pocket; *C *– three amiloride molecules bind at the interfaces to membrane-spanning helices; *D *– two amiloride molecules form a dimer, which binds in the acidic pocket

In addition to this peptide toxin, a complex formed between ASIC1a and amiloride has been resolved within the same structure. Three amiloride molecules reside in the upper portion of the channel pore, at the interfaces between subunits (*Fig. 4C*). Their charged groups are exposed to the pore lumen. On the other hand, mutagenesis data are indicative of deeper location of the amiloride binding sites in the pore [75]. Kellenberger et al. [75] put forward a hypothesis that the binding sites found in the X-ray crystallographic structure represent the intermediate position of amiloride; one molecule may go deeper, thus sterically blocking the channel. The second binding site of amiloride is located in the acidic pocket (*Fig. 4D*). Two molecules form a dimer, which is stabilized by stacking interactions between aromatic groups and the oppositely oriented guanidine groups. The functional role of this binding site is still unknown, but it seems likely that it is related to the ability of amiloride to activate ASICs [33].

The binding site of psalmotoxin identified by Baconguis et al. [73] significantly overlaps with the binding region of MitTx. However, the middle loop of a psalmotoxin molecule enters the acidic pocket, where the positively charged toxin residues directly interact with the acidic residues of the receptor (*Fig. 4B*). In other words, the stabilization mechanisms of the open state mediated by MiTtx and the desensitized state mediated by psalmotoxin differ fundamentally: psalmotoxin affects the proton receptor, whereas MiTtx affects the executive mechanism of activation.

The putative binding sites of other ASIC ligands can be derived from indirect evidence by analyzing the data on the mechanisms of action, the effect of point mutations, and competition with ligands whose binding sites are known. For example, the findings on the common binding regions of MitTx and psalmotoxin are consistent with the data on the mutually exclusive effect of these toxins [56]. It has been reported most recently [76] that mambalgins also bind near the acidic pocket (like psalmotoxin does).

Binding of several other types of ligands can be related to psalmotoxin. Thus, Duan et al. [45] analyzed the simultaneous action of spermine and psalmotoxin and demonstrated that these two compounds can compete for the common binding site, although they modulate the ASIC1a function in opposite directions. There also exists competition between psalmotoxin and calcium [54]; furthermore, calcium is known to compete with protons [43, 77]. Competition with palmotoxin was also shown for dynorphin [51]. Based on these data, it is fair to assume that the ligands affecting the pH dependence of activation and steady-state desensitization of ASIC1a bind within the acidic pocket of the receptor and have a direct impact on its functional components. Since the acidic pocket contains several negatively charged amino-acid residues, a specific mode of action for each ligand can be determined by specific interactions with this area.

However, some data is not entirely consistent with this concept. First, as noted above, the acidic pocket is not the only proton-binding site required for channel activation [70, 71]. The substitution of amino-acid residues in the palm domain affects desensitization and activity of a number of ligands. In their recent paper [78], Besson *et al*. systematically studied the mutual effects of GMQ, amiloride, psalmotoxin, and mambalgine. They found that the impact of GMQ and mambalgine on the ASIC1a activation is fully additive, suggesting that their mechanisms of action are independent. On the contrary, the effects of GMQ and psalmotoxin on steady-state desensitization are not independent, since there exists a negative cooperativity between them. Hence, the question pertaining to the exact binding sites of the ligands affecting the activation and desensitization of ASIC1a remains open and requires further research.

## CONCLUSIONS

The data on the effect of various ligands on ASIC1a suggest three main modes of action: blocking of the channel pore, shifting of the dependence of activation on pH, and shifting of the dependence of desensitization on pH. Many ligands simultaneously affect the latter two characteristics. Effects of this type are probably mediated by ligand binding to some extracellular site, which controls the activation and desensitization characteristics of ASIC1a. Although the ligand-induced shifts in these curves usually do not exceed 0.2–0.5 pH units, they have a substantial impact on the function of the channel, since ASIC1a activation takes place in a pH range between 7.0 and 5.0, while steady-state desensitization develops at pH between 7.5 and 7.0. Such characteristics significantly limit the possibility of ASIC1a functioning under physiological conditions. In particular, even insignificant acidification causes desensitization and loss of channel function. From this point of view, it seems promising to search for endogenous ligands that shift desensitization towards more acidic pH values and, contrariwise, shift activation towards smaller acidification. In the presence of such ligands, ASIC1a could be activated by small acidification of the external medium that occurs upon synaptic transmission. In this case, moderate acidification of the medium would not lead to desensitization, but rather enhance the response and the contribution of ASIC1a to neuronal excitability. If we take into account the fact that elevated activation of ASIC1a can have a beneficial impact on the course of many pathological processes, detection of ligands that potentiate ASIC1a acquires a clinical significance.
